# Aging with HIV: Increased Risk of HIV Comorbidities in Older Adults

**DOI:** 10.3390/ijerph19042359

**Published:** 2022-02-18

**Authors:** Rifqah Abeeda Roomaney, Brian van Wyk, Victoria Pillay-van Wyk

**Affiliations:** 1 Burden of Disease Research Unit, South African Medical Research Council, Cape Town 7505, South Africa; victoria.pillay-vanwyk@mrc.ac.za; 2 School of Public Health, University of the Western Cape, Cape Town 7535, South Africa; bvanwyk@uwc.ac.za

**Keywords:** HIV, comorbidity, multimorbidity, aging, South Africa

## Abstract

With improved access to antiretroviral treatment (ART), adults with HIV live longer to reach older age. The number of older adults living with HIV is increasing steadily, giving rise to a new population of interest in HIV research and for invigorated considerations in health service delivery and policy. We analysed the profile of comorbidities in older people (50 years and older) living with HIV in South Africa. We conducted a secondary analysis of all individuals over 15 years who tested HIV positive in the Fifth South African National HIV Prevalence, Incidence, Behaviour and Communication Survey, 2017. We conducted multivariate logistic regression to determine the factors associated with having HIV comorbidity using Stata 15.0 software. We entered 3755 people living with HIV into the analysis, of whom 18.3% (*n* = 688) were 50 years or older. Older adults had four times greater odds (OR = 4.7 (3.1–7.0)) of having an HIV comorbidity compared to younger adults. Being female (OR = 1.6 (1.1–2.4)) and living in an urban area (OR = 2.6 (1.8–3.7)) increased the odds of HIV comorbidity. Older adults with HIV require comprehensive health care to deal with multimorbidity, to maximise the benefits gained by advances in HIV therapies.

## 1. Introduction

South Africa has a generalised and mature HIV epidemic [1]. In 2017, the national HIV prevalence was estimated at 14%, which translated to approximately 7.8 million people living with HIV (PLWH). South Africa has the largest public antiretroviral therapy programme (ART) [1], with 5.6 million people on ART in 2020 [2]. South Africa has made some progress towards achieving The Joint United Nations Programme on HIV/AIDS (UNAIDS) 90–90–90 targets (that by 2020, 90% of all people living with HIV should know their status, that 90% of people diagnosed with HIV infection should receive treatment and that 90% of all people receiving treatment should have viral suppression [3]). In 2020, it was estimated that 92% of PLWH in South Africa knew their status [2], of which 75% were on ART and of which 92% on ART were virally suppressed [4]. It was further estimated that 70% of all PLWH in South Africa were on ART in 2020 [4]. It is envisaged that the number of PLWH on ART is likely to continue to grow as the country endeavors to meet ART coverage targets [5]. Improved access to ART, coupled with advances in ART regimens, have enabled PLWH to live longer lives [6], effectively changing HIV into a life-long chronic condition [7]. It has been noted that South Africa has a quadruple burden of disease [8], which includes a growing burden of non-communicable diseases (NCDs) [8]. A decade prior, researchers were predicting the negative impacts associated with the collision of these two epidemics in South Africa [9]. More recently, studies have found a high and overlapping prevalence of disease conditions such as HIV, tuberculosis and NCDs in both community settings [10,11,12] and health facilities in the country [13].

Several studies have shown that PLWH has a higher prevalence of multimorbidity and comorbidity compared to the general population due to premature ageing, side effects of ART and biological effects of HIV infection [14,15,16,17,18]. The most prevalent HIV comorbidities are cardiovascular diseases, cancers, diabetes, dyslipidaemia, chronic renal disease and hepatitis B and hepatitis C [14]. Multimorbidity in PLWH drives healthcare costs and the number of hospitalisations, as shown in a case-control study in France where the mean total cost of hospitalisation was six times higher in PLWH compared to matched controls [14].

The number of older adults (aged over 50 years) living with HIV is increasing globally, which naturally leads to an increase in the prevalence of HIV comorbidities [19,20]. This increase is mainly due to earlier initiation on ART compared to the pre ‘Test and Treat’ era, which improved chances for survival for HIV populations [21]. The resultant longer duration of ART exposure has also been linked to increased hypertension risk [22,23,24]. In addition, it is reported that HIV infections also occur at older ages [25,26], and such persons may have already developed NCDs [22,27]. 

Older PLWH experience increased prevalences of non-AIDS illnesses such as cardiovascular disease, malignancies, osteoporosis, cognitive impairment, frailty and disability [28]. Another study found that rates of NCDs were higher among older adults with HIV compared to younger adults with HIV [29]. Moreover, there are still many uncertainties in treating HIV in older adults when the individual has comorbidities [22]. For example, older people with HIV have higher risks of hospitalisation due to adverse events from polypharmacy [30]. In general, older adults face additional barriers to care such as patronising and ageist communication by health care professionals, exclusion from clinical trials and low income associated with retirement [31].

While the number of older adults living with HIV is expected to increase, little is known about HIV and ageing in low and middle-income countries, especially sub-Saharan Africa [22]. Such information is critical for the provision of integrated care for older adults at the primary care level in HIV endemic settings. The current paper reports on the profile and patterns of comorbidities in older adults living with HIV in South Africa.

## 2. Materials and Methods

### 2.1. Study Design and Aim

The South African National HIV Prevalence, Incidence, Behaviour and Communication Survey 2017 (SABSSM 2017) is the fifth iteration of the national household survey of HIV that aimed to estimate the HIV incidence and prevalence in a representative sample of South Africans as well as self-reported health conditions and health behaviours [1]. The methods used in the survey are described in full in Simbayi, Zuma [1]. The main aim of the survey was to provide surveillance information for monitoring trends in HIV incidence, prevalence and related behaviours, in addition to describing self-reported disease conditions.

### 2.2. Sample and Data Collection 

All people in South Africa were eligible to participate in SABSSM 2017 survey. The survey employed a multi-stage, stratified random cluster sampling approach to identify eligible households for inclusion. There were several questionnaires aimed at different age groups. Youth and adults were invited to complete a questionnaire and were asked several questions about their health, risk perceptions and behaviours. Participants were asked if they ‘…*currently have any of the following illnesses*?’. We included five self-reported health conditions for this study: cancer, diabetes, heart disease, hypertension/high blood pressure and Tuberculosis (Supplementary Material Table S1). These disease conditions were included based on common comorbid conditions [32,33]. If the participant consented to an HIV test, a finger prick was used to obtain a dry blood spot sample. The dry blood spot samples were then transported to accredited laboratories for linked anonymous HIV testing. The samples were tested for HIV antibodies and if the samples tested positive using the first two immunoassays, a third test was conducted for confirmation [1]. 

### 2.3. Ethics Considerations

The SABSSM 2017 data were ethically obtained from participants and described in full here [1]. For the purpose of the current data analysis, access to anonymised data was obtained from the online portal, the HSRC Research Data Service. Ethics clearance for the analysis of secondary data (as part of the first author’s Ph.D. study) was granted by the Biomedical Science Research Ethics Committee of the University of the Western Cape (BM20/5/8). 

### 2.4. Data Analysis

Data cleaning and statistical analysis were conducted using Stata 15.0 software (Stata Corporation, College Station, TX, USA). The Stata survey set of commands (‘svy’) was used to account for the complex survey design.

The analysis was restricted to people over the age of 15 years that tested HIV positive. We created an age variable that separated participants into two groups: those aged 15–49 years (‘under 50s’) and those aged 50 years and above (‘over 50s’). Among the sample, we also examined the distribution of people who had HIV only and those who had HIV and at least one other disease (i.e., comorbidity). Therefore, we created an indicator variable that counted the number of diseases present in each individual (excluding HIV). 

We used frequencies to display categorical data. The bivariate associations between age category and other variables were tested using Chi-square tests. Multivariate logistic regression was conducted to test the associations of factors with participants having HIV only or having HIV and one or more comorbidity. We included factors based on characteristics that are often associated with multimorbidity and the data available [34]. These factors were: age, sex, locality, educational attainment, employment status and smoking status. We estimated the crude odds ratios and then combined factors in a multivariate model. Model-checking was performed on the unweighted model. We assessed the model for influential observations using the Pearson residuals, deviance residuals and Pregibon’s leverage [35]. Crude and adjusted odds ratios are reported with 95% CIs, and we considered *p*-values of less than 0.05 to be statistically significant.

## 3. Results

### 3.1. Sample Description

**Figure 1:**
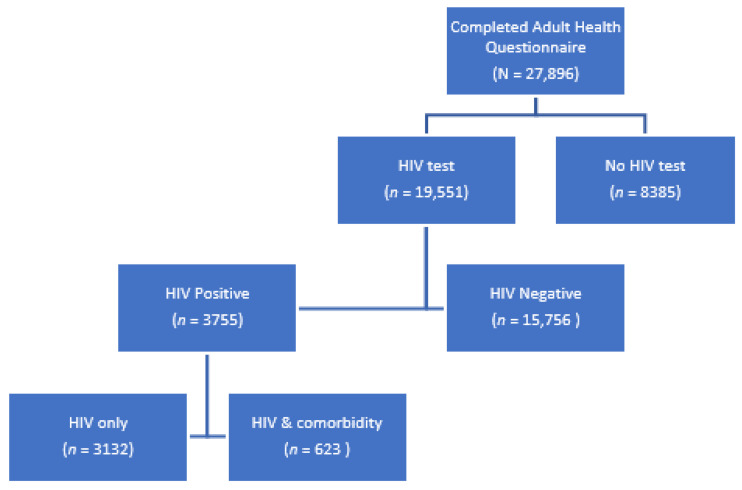
HIV testing in the SABSSM 2017 sample.

Of the 27,896 youth and adults that completed the Adult Health Questionnaire, 69.9% (*n* = 19,511) had results for an HIV test (Figure 1). Of these tested, 19.3% (*n* = 3755) were HIV positive. The majority had HIV only (83.4%, *n* = 3132).

Table 1 describes the sociodemographic characteristics of the sample population with HIV by age sub-groups (under 50 years versus 50 years and over). Older adults (50 years or older) living with HIV constituted 18.3% (*n* = 688) of the sample. Most participants were female (73.4%, *n* = 2754). More than a third of the respondents were based in Kwa-Zulu Natal province (38.4%, *n* = 1442). Employment was generally low in the sample (27.9%), and it was statistically significantly lower in the older age group compared to the younger age group (*p* < 0.001). Most respondents (70.6%) had completed secondary education, and the younger group of people had significantly higher levels of educational attainment than the older group of people (*p* < 0.001).

### 3.2. Prevalence of Comorbidities in Those with HIV

A large proportion of the study population only had HIV (82.2%, 95% CI: 80.0–84.3%), with large variation between younger and older adults (87.0 (84.6–89.0 vs. 55.9 (49.7–61.9)) (Table 2). Almost 15% of the study population had HIV and one other disease, with a difference among the under 50s compared to the over 50s (11.1% versus 35.5%). A comorbidity was present in 18% (95% CI: 15.7–20.0%) of the HIV positive population, and was far more prevalent in older adults (44.1%, 95% CI: 38.1–50.3%) compared to younger people (13.0%, 95% CI: 11.0–15.4%). 

**Figure 2:**
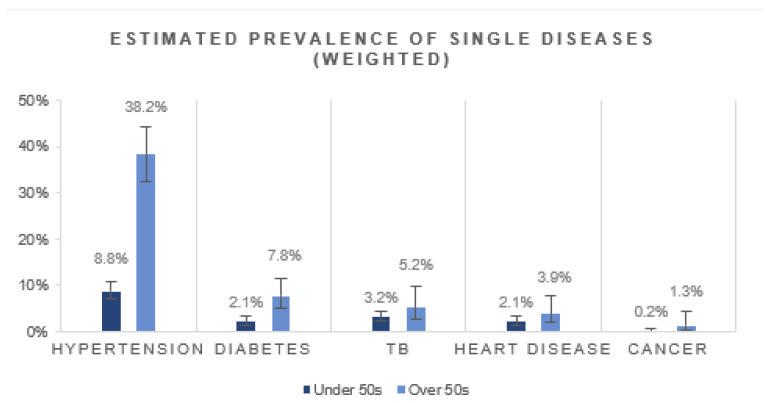
Estimated prevalence of single disease conditions in HIV-positive people by age group.

Of the diseases assessed, hypertension was estimated to be the most prevalent comorbidity among people living with HIV (13.3%, 95% CI: 11.5–15.3%). Hypertension was more prevalent among people over the age of 50 years compared to those under the age of 50 years (Figure 2). Estimated TB was the next most prevalent disease (3.5%, 95% CI: 2.6–4.8), followed by diabetes (3.0%, 95% CI: 2.1–4.2), heart disease (2.3%, 95% CI: 1.5–3.5) and cancer (0.4%, 95% CI: 0.1–0.9). All of the diseases were estimated to be more prevalent among the older group. Generally, females had a higher disease prevalence for hypertension, diabetes, heart disease and cancer (Supplementary Material Figure S1).

### 3.3. Factors Associated with Having HIV Comorbidity

We assessed factors possibly associated with having an HIV comorbidity compared to only having HIV (Table 3). For the adjusted model, outliers were dropped, and the model was refitted due to the model having limited ability in predicting comorbidity in women 50 years and older that were living in rural areas with secondary education and did not drink alcohol (Supplementary Material Table S2, Supplementary Material Figure S2).

Those belonging to the age group 50 years and older had almost five times the odds of having an HIV comorbidity compared to those in the under 50 years age group (OR: 4.7, (95% CI: 3.1–7.0)). Females had almost double the odds of having comorbidity compared to males (OR: 1.6, (95% CI: 1.1–2.4)). Those living in urban areas were more likely to have an HIV comorbidity compared to those living in rural areas (OR: 2.6, (95% CI: 1.8–3.7)). Being employed (OR: 0.6, 95% CI: 0.4–0.9) was protective against having a comorbidity.

## 4. Discussion

This analysis of national survey data showed that PLWH over 50 years were more than twice as likely to have an HIV comorbidity compared to PLWH under 50 years. Our study generally confirms other research findings that older adults with HIV had high rates of chronic diseases when compared to younger adults with HIV in South Africa [29]. A survey of HIV amongst educators in South Africa reported a higher overall prevalence of HIV comorbidities compared to our study (36.9% versus 17.8%) [36]. The educator study, however, reports similar estimates of older adults with HIV comorbidities to our study (36.9% versus 44.1%). The differences in estimates of HIV comorbidity could be due to differences in the populations surveyed and the way that chronic diseases were ascertained. The inclusion of employed educators would have automatically excluded young individuals (i.e., youth aged 15–20 years). Moreover, their population of interest differed from ours as they were employed and more likely to have tertiary education, whereas our study was a household survey with few participants employed or possessing tertiary education. The way that chronic diseases were defined between the two studies also differed. In the educator study, disease conditions were included if the educator was diagnosed in the last five years, whereas we included disease conditions that the participant ‘currently’ had. Despite the differences, all three studies report high levels of HIV comorbidities and also that hypertension was the most common HIV comorbidity. The high prevalence of HIV-hypertension comorbidity is also corroborated by other local studies (e.g., hospital admissions in the North West Province [37]) as well as studies in the region (e.g., a retrospective analysis of routine medical records conducted in Malawi [38]).

Our multivariate analysis confirmed that being over the age of 50 years was associated with a nearly five-fold increase in having HIV comorbidity. Being female and living in an urban area also increased the odds of having a comorbidity. In contrast, employment reduced the odds of having a comorbidity.The results of our regression were similar to the educator study [36] in that they found that women, older adults and living in urban areas increased the odds of having an HIV comorbidity. While women and older adults are known to have higher prevalences of comorbidity or multimorbidity [39], it is unclear why urban residents would have higher levels of comorbidities. This could be related to the unmet need for care in rural areas (i.e., people in rural areas could be unaware they have an NCD and thus it is underreported) [40,41], or it could be due to nutritional shifts and reduced physical activity associated with urbanisation [42].

Our study indicates that a large proportion of older PLWH is managing at least one other comorbid disease condition. In addition, the prevalent HIV comorbidities are not necessarily AIDS-related opportunistic infections traditionally associated with HIV. Of the diseases we assessed, hypertension and diabetes were the most prevalent diseases in older PLWH, confirming the shift towards chronic comorbidities usually associated with ageing. As the ART programme expands and more PLWH reach older ages, the prevalence of NCD comorbidities can be expected to rise, which has the potential to negatively impact service delivery in South Africa. Concerningly, the pattern of comorbidities was also similar among younger PLWH (although TB was more prevalent than diabetes). This means that younger PLWH will have to be on medication for HIV and another disease, possibly for life. This could impact their quality of life and long-term adherence negatively while also increasing the cost of treatment per individual in the public health sector. 

Our study is based on 2017 data and reiterates that the integration of HIV and NCD services at the primary care level is urgent. The need for integrated care was acknowledged in the health policy space almost a decade ago (in 2011) when the South African government adopted an Integrated Chronic Disease Management (ICDM) model [43]. However, the implementation of this policy and concomitant regulations at the service delivery level has been largely left unattended. As new diseases continue to emerge, it is imperative that the health system aggressively tackle HIV and its comorbidities. The UNAIDS 95-95-95 testing, treatment and viral suppression targets highlight the need to ensure accessibility, availability and affordability of safe, quality-assured medicines to prevent, diagnose and treat HIV infections, co-infections and comorbidities [44]. More research is needed on ART in older people as they tend to be underrepresented or excluded from studies on ART [45]. There are multiple toxicities to consider—such as specific agents or drug classes that are known to have adverse bone and renal effects or increase cardiovascular risk [45]. More research is also needed on adherence, toxicity and the influence of comorbidities [46]. 

This study was limited to the data available in the original survey. It is also limited by the use of self-reported disease conditions for comorbidities. Self-reported disease conditions could be underestimated if the person is unaware they have a disease or if they choose not to disclose the disease. For example, in a recent comparison of two national South African surveys that took hypertension measurements, the hypertension prevalence among the 15 years and older population were estimated to be 38.4% in the 2012 South African National Health and Nutrition Examination Survey (SANHANES) and 48.2% in the 2016 South African Demographic and Health Survey (SADHS) [47]. Our estimate of hypertension in this population was much lower (13.3%), which could be attributed to the use of self-reported data. Similarly, the 2016 SADHS [48] also estimated a higher prevalence of measured diabetes (13% of women, 8% of men) compared to our 3% of self-reported diabetes. This indicates that the true prevalence of comorbidities among PLWH could be much higher than what was estimated in this study. 

## 5. Conclusions

This study provides evidence of the extent of comorbidity in older and younger PLWH from the analysis of the 2017 SABSSM survey. The information from this study (including common comorbidities) could be used to inform and motivate service integration for PLWH. Information on common comorbidities could also be used to inform screening efforts at the primary care level and target older people, women and people living in urban areas. Given these high levels of comorbidity (particularly the 44% among older PLWH), the pressing need for the implementation of integrated care of NCD and HIV services is clear. HIV cannot be treated in a silo, especially as more people are expected to be initiated on ART in an effort to reach the ambitious UNAIDS 95–95–95 targets, whereby 95% of people diagnosed with HIV should be on ART treatment in 2030. Prevention efforts are needed to ensure that the prevalence of HIV comorbidities is reduced where possible. Regular screening for NCD comorbidities is also essential to ensure optimal and efficient treatment for PLWH. 

## Figures and Tables

**Table 1:** Sociodemographic characteristics of adults who tested HIV positive by age (under 50 years vs. 50 years and older), SABSMM 2017.

Variable	% (*n*)			*p*-Value *
	Total (N = 3755)	Under 50s (*n* = 3066)	Over 50s (*n* = 688)	
Age (Median years and IQR)	36 (29–46)	33 (28–40)	56 (53–61)	-
Female	73.4 (2754)	73.9 (2266)	80.0 (487)	0.115
Urban location	45.6 (1712)	45.7 (1402)	45.1 (310)	0.750
Province				<0.001
Eastern Cape	7.4 (277)	7.5 (230)	6.8 (47)	
Free State	4.7 (177)	4.2 (130)	6.8 (47)	
Gauteng	13.9 (523)	13.9 (427)	14.0 (96)	
KwaZulu-Natal	38.4 (1442)	38.6 (1184)	37.5 (258)	
Limpopo	5.1 (193)	4.5 (137)	8.1 (56)	
Mpumalanga	15.6 (585)	16.0 (490)	13.7 (94)	
Northern Cape	3.4 (126)	3.5 (108)	2.6 (18)	
North-West	7.2 (269)	7.2 (220)	7.1 (49)	
Western Cape	4.3 (163)	4.6 (140)	3.3 (23)	
Employed/Self-employed	27.9 (987)	28.9 (836)	23.6 (151)	0.007
Education level				<0.001
Primary or less	23.9 (688)	18.4 (446)	52.9 (241)	
Secondary complete	70.6 (2036)	76.0 (1844)	42.1 (192)	
Tertiary	5.6 (161)	5.7 (138)	5.0 (23)	

* Chi-square tests.

**Table 2:** Prevalence of comorbidities in adults living with HIV.

Number of Comorbidities	Weighted % (95% CI)		
	Total	Under 50s	Over 50s
HIV only	82.2 (80.0–84.3)	87.0 (84.6–89.0)	55.9 (49.7–61.9)
1 comorbidity	14.8 (12.9–16.9)	11.1 (9.2–13.2)	35.5 (29.9–41.6)
2 comorbidities	2.4 (1.7–3.4)	1.6 (1.0–2.6)	7.2 (4.6–11.0)
3 + comorbidities	0.5 (0.3–1.0)	0.4 (0.1–0.9)	1.4 (0.4–4.1)
HIV and comorbidity	17.8 (15.7–20.0)	13.0 (11.0–15.4)	44.1 (38.1–50.3)

**Table 3:** Factors associated with having HIV comorbidity.

Variable	Unadjusted Odds Ratios (95% CI)	Adjusted Odds Ratios (95% CI)
Age over 50 years (Reference: Under 50s)	5.3 (3.8–7.3)	4.7 (3.1–7.0)
Sex (Reference: Male)	1.4 (1.0–2.0)	1.6 (1.11–2.4)
Urban (Reference: Rural)	1.9 (1.4–2.5)	2.6 (1.8–3.7)
Education (Reference: Primary)		
Secondary	0.6 (0.4–0.9)	0.7 (0.5–1.1)
Tertiary	1.1 (0.5–2.7)	1.4 (0.5–3.6)
Employed (Reference: Not employed)	0.6 (0.4–0.9)	0.6 (0.4–0.9)
Current alcohol use (Reference: No current alcohol use)	1.0 (0.7–1.4)	1.1 (0.8–1.7)

## Data Availability

The datasets analysed for this study can be found in the HSRC Research Data Service (https://repository.hsrc.ac.za/handle/20.500.11910/15468). Accessed on 10 February 2022.

## Supplementary Materials

The following supporting information can be downloaded at: https://www.mdpi.com/article/10.3390/ijerph19042359/s1, Figure S1: Single disease prevalence by sex (weighted); Figure S2: Model fit graphs; Table S1: Self-reported disease questions; Table S2: Model with outliers included.
