# Supplementary material for: Aging with HIV: Increased Risk of HIV Comorbidities in Older Adults
Source: Int J Environ Res Public Health. 2022 Feb 18;19(4):2359. doi: 10.3390/ijerph19042359 (PMC8872228; doi:10.3390/ijerph19042359)

International Journal of
Environmental Research and Public Health

MDPI

Table S1. Self-reported disease questions.

Disease condition	Question
Cancer	Do you currently have any of the following illnesses? (Diagnosed with illness) Cancer
Diabetes or blood sugar	Do you currently have any of the following illnesses? (Diagnosed with illness) Diabetes
Heart disease	Do you currently have any of the following illnesses? (Diagnosed with illness) Heart disease
High blood pressure	Do you currently have any of the following illnesses? (Diagnosed with illness) Hypertension / high blood pressure
HIV	Do you currently have any of the following illnesses? (Diagnosed with illness) HIV
Tuberculosis	Do you currently have any of the following illnesses? (Diagnosed with illness) Tuberculosis/ TB

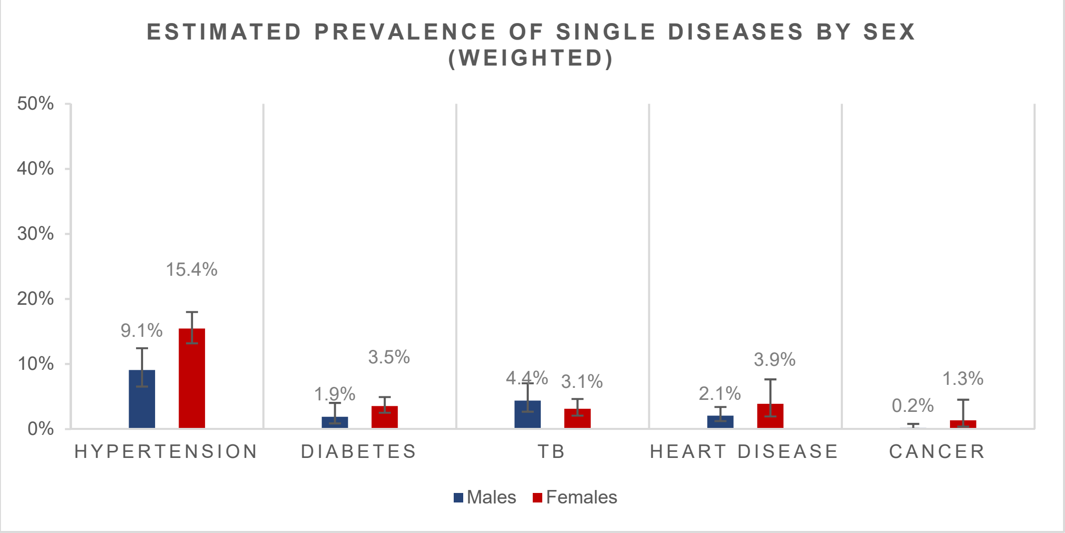

Figure S1. Single disease prevalence by sex (weighted).

Table S2. Model with outliers included

**Variable**	**Adjusted Odds ratios (95% CI)**
Age over 50 years (Reference: Under 50s)	4.68 (3.67 - 5.97)
Sex (Reference: Male)	1.16 (0.91 – 1.49)
Urban (Reference: Rural)	2.05 (1.66 - 2.54)
Secondary	0.80 (0.63 - 1.02)
Tertiary	1.11 (0.70 - 1.80)
Employed (Reference: Not employed)	0.78 (0.61 - 0.99)
Current alcohol use (Reference: No current alcohol use)	0.93 (0.71 - 1.21)

Figure S2. Model fit graphs.

Standardized Pearson residuals

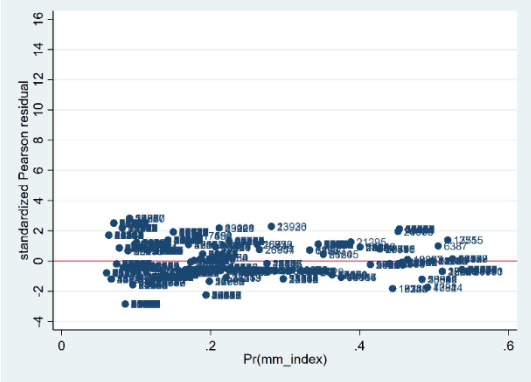

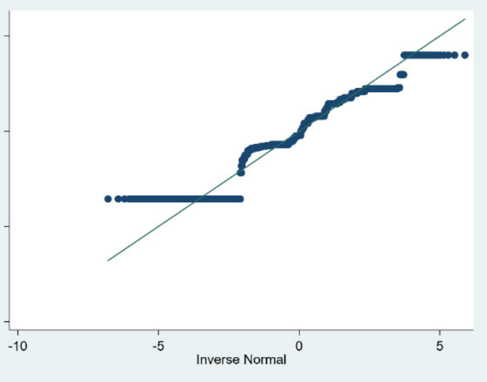

Leverage

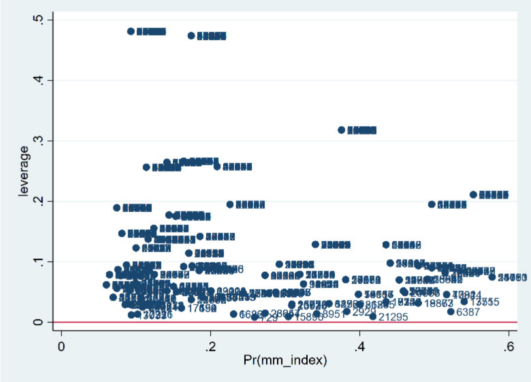

Pregibon's dbeta

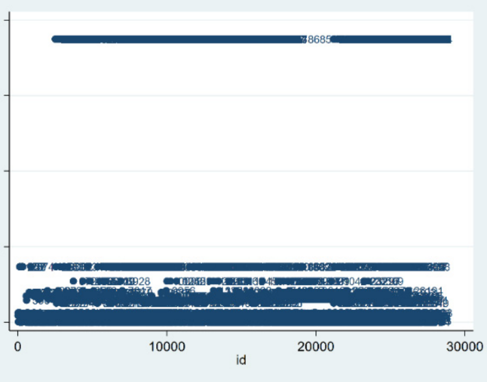

Deviance residuals

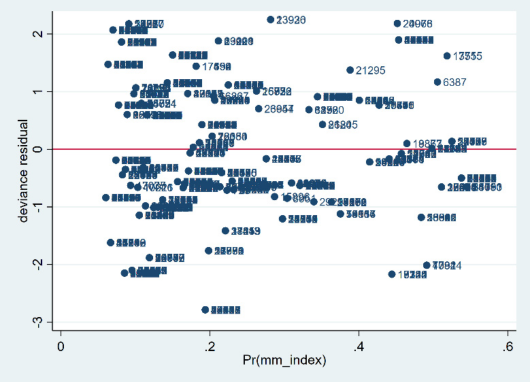

Supplement: Supplementary file 1 [file ijerph-19-02359-s001.zip › ijerph-1565470-supplementary.pdf]
